# Pravastatin Protects Against Avascular Necrosis of Femoral Head via Autophagy

**DOI:** 10.3389/fphys.2018.00307

**Published:** 2018-04-09

**Authors:** Yun Liao, Ping Zhang, Bo Yuan, Ling Li, Shisan Bao

**Affiliations:** ^1^Department of Pharmacy, Tongren Hospital, Shanghai Jiao Tong University School of Medicine, Shanghai, China; ^2^Department of Pharmacy, Shanghai Tenth People's Hospital, Tongji University, Shanghai, China; ^3^Discipline of Pathology, Charles Perkin Center, School of Medical Sciences and Bosch Institute, University of Sydney, Sydney, NSW, Australia

**Keywords:** autophagy, avascular necrosis of the femoral head, pravastatin, endothelial progenitor cells, AMPK, mTOR, LKB1

## Abstract

Autophagy serves as a stress response and may contribute to the pathogenesis of avascular necrosis of the femoral head induced by steroids. Statins promote angiogenesis and ameliorate endothelial functions through apoptosis inhibition and necrosis of endothelial progenitor cells, however the process used by statins to modulate autophagy in avascular necrosis of the femoral head remains unclear. This manuscript determines whether pravastatin protects against dexamethasone-induced avascular necrosis of the femoral head by activating endothelial progenitor cell autophagy. Pravastatin was observed to enhance the autophagy activity in endothelial progenitor cells, specifically by upregulating LC3-II/Beclin-1 (autophagy related proteins), and autophagosome formation *in vivo* and *in vitro*. An autophagy inhibitor, 3-MA, reduced pravastatin protection in endothelial progenitor cells exposed to dexamethasone by attenuating pravastatin-induced autophagy. Adenosine monophosphate-activated protein kinase (AMPK) is a key autophagy regulator by sensing cellular energy changes, and indirectly suppressing activation of the mammalian target of rapamycin (mTOR). We found that phosphorylation of AMPK was upregulated however phosphorylation of mTOR was downregulated in pravastatin-treated endothelial progenitor cells, which was attenuated by AMPK inhibitor compound C. Furthermore, liver kinase B1 (a phosphorylase of AMPK) knockdown eliminated pravastatin regulated autophagy protein LC3-II in endothelial progenitor cells *in vitro*. We therefore demonstrated pravastatin rescued endothelial progenitor cells from dexamethasone-induced autophagy dysfunction through the AMPK-mTOR signaling pathway in a liver kinase B1-dependent manner. Our results provide useful information for the development of novel therapeutics for management of glucocorticoids-induced avascular necrosis of the femoral head.

## Introduction

There has been an increased prevalence of avascular necrosis of the femoral head (ANFH), attributed to the widespread use of glucocorticoids (GCs) in transplant recipients and patients with rheumatologic diseases (Mont et al., 2015). It is reported that ANFH often co-exists with bone degenerative and vascular diseases (Weinstein et al., 2010). Recent studies demonstrate that endothelial injury is more severe in GCs-induced ANFH than that from non-GCs users (El Zaoui et al., 2015). GCs regulate the process of adipogenesis in bone marrow stroma, which contributes to hypertrophy and hyperplasia of adipocytes resulting in increased intraosseous pressure and decreased blood flow rate (Miyanishi et al., 2002). Endothelial integrity is subsequently compromised, leading to circulatory obstruction and necrosis of the femoral head in GCs user patients (Weinstein, 2012). Excess GCs exposure has been previously linked with activation of cellular apoptosis, precipitating expanding investigations into the role of apoptosis in the development of ischemic osteonecrosis (O'Brien et al., 2004).

Endothelial progenitor cells (EPCs) are a group of multi-progenitor cells capable of self-renewal and differentiation into endothelial cells (Asahara et al., 1997). EPCs play a critical role in vascular endothelium integrity, through vascular repair and regeneration (Liu et al., 2011). Reduced numbers and impaired function of EPCs have been correlated with increased risk for ANFH (Feng et al., 2010). Furthermore, Laane et al., reported that dexamethasone (Dex) induced lymphoid cell death through the induction of autophagy before apoptosis (Laane et al., 2009), suggesting the existence of other important cell death pathways that may contribute to the vascular pathophysiology of ANFH in EPCs.

Autophagy classically contributes to cellular homeostasis and adaptation to stress, through a complex process that degrades intracellular components by forming autophagosomes (Eisenberg-Lerner et al., 2009). Interestingly, depending on cell types and conditions, autophagy may also play an opposing role in cellular survival (Codogno and Meijer, 2005). Adaptive autophagy may provide cytoprotection in energy starvation or environmental stress conditions (Hamacher-Brady et al., 2006), however prolonged autophagy activity precipitates apoptosis (Azad et al., 2008). The relationship between autophagy in the GCs-induced vascular osteonecrosis and EPCs in ANFH therefore remains to be explored.

Pravastatin, a member of the statin family of 3-hydroxy-3-methylglutaryl-coenzyme A reductase inhibitors, is widely used for the management of acute myocardial infarction and unstable angina pectoris by reducing risk of death and major cardiovascular events (Hague et al., 2016). In addition to cardiac disease management, statins can be used as therapeutic agents for the treatment of hypercholesterolemia (Istvan and Deisenhofer, 2001). The beneficial effects of statins have been further suggested to extend beyond cholesterol reduction, to include vascular inflammation suppression, oxidative stress attenuation, and endothelial dysfunction amelioration involved in angiogenesis (Wilson et al., 2002). Several signaling pathways have been implicated in these protective roles of statins, including AMP-activated protein kinase (AMPK)/mTOR pathway, phosphatidylinositol 3-kinase/Akt pathway, and mitogen-activated protein kinase/extracellular signal-regulated kinase pathway (Kureishi et al., 2000; Dong et al., 2011; Xu et al., 2011). More recently, autophagy induction has been suggested as a novel process by which statins regulate cell survival (Araki et al., 2012).

It is currently unclear if pravastatin can reduce ANFH development *in vivo*, or more specifically, which protective signaling pathway(s) are activated by pravastatin. Thus, the current study demonstrates pravastatin-regulated autophagy of EPCs in Dex-induced ANFH *in vivo*. This modulation on autophagic process may contribute to the protective effect of pravastatin against GCs injury in ANFH. Our investigation would provide some insight in both prevention and/or management of steroids induced femoral head necrosis for clinical applications.

## Materials and methods

### Animals

Adult male Wistar rats (8–10 w, 250–300 g) were purchased from Shanghai Experimental Animal Center, Chinese Academy of Sciences. The animals were housed 5 per cage in controlled conditions (temperature: 21 ± 2°C and lighting: 8:00–20:00) and received standard animal chow and water *ad libitum*. All animals received human care in compliance with Laboratory Animal Care and Use Guideline of the Shanghai Tenth People's Hospital (SYXK 2014-0026), and the Guide for Care and Use of Laboratory Animals published by the National Institutes of Health (SCXK 2013-0016).

### Animal modeling and groups

Rats were weighed after feeding for 1 week and randomly divided into the following three groups: Control group (*n* = 20), ANFH group (*n* = 30) and ANFH-Pravastatin (ANFH-PS) group (*n* = 30). Rats in ANFH and ANFH-PS groups were intravenously injected with dexamethasone (Kerachian et al., 2011) (Dex, 0.5 mg/kg/d × 30 d, D1756-1G, Sigma-Aldrich, St Louis, MO, USA). Meanwhile, the control and ANFH groups were treated over 30 days with vehicle (0.5% CMC) once daily *p.o*., while the ANFH-PS group was treated with pravastatin 4 mg/kg once daily, *p.o*. The dosage of pravastatin used for the experiment was calculated by converting human dose used in main indication to rodent dose based on the following criteria: rat dose (mg/kg) = [human dose (mg/kg) × 60 kg × 0.018]/0.2 (Nair and Jacob, 2016). On day 30, rats in the three groups were anesthetized with a 3-min inhalation of ether as described (Zhang et al., 2013), and the femoral heads were harvested or EPCs were isolated from bone marrow (Figure S1).

### Magnetic resonance imaging (MRI) scan and biomechanical examination

MRI is considered an imaging method with the highest sensitivity and specificity for ANFH early diagnosis and staging evaluation (Fujioka et al., 2001). After the final Dex application, MRI was performed for the bilateral proximal femora, using a 3.0-T superconducting system (GE, Fairfield, Connecticut). Short-tau inversion recovery (STIR) imaging parameters were follows: repetition time (TR) = 6200 ms; echo time (TE) = 30 ms; FOV = 16 × 16; matrix = 256 × 146; excitation flip angle = 90^0^. STIR high signal which was identified as fat appearance and potential femoral head osteonecrosis (Fujioka et al., 2001). The MRI results of early ANFH were compared among the groups (*n* = 5 per group).

Bone mineral density was determined by microcomputed tomography (eXplore Locus SP; GE cop; scan resolution, 14 mm; voltage, 80 kV; current, 80 mA). Compressive strength was assessed by a universal mechanical testing system (distortion measurement accuracy, 0.005 mm; load measurement accuracy, 1N; compressive strength = P/A; *n* = 5 per group).

### Bone marrow-derived EPCs (BM-EPCs) culture, drug treatments, and transfection

Rat BM-EPCs were isolated and cultured according to described technique (Chen et al., 2013). After 7 days of culturing, medium was replaced with Dex (1 μM) alone, or co-treated with pravastatin (1 μM) for 12, 24, and 48 h. Additional reagents used in this study included rapamycin (10 μM, autophagy inductor, Sigma-Aldrich, 37094), 3-MA (5 mM, autophagy inhibitor, Sigma-Aldrich, M9281) and compound C (10 mM, AMPK inhibitor, Calbiochem, San Diego, CA, USA, 171260). liver kinase B1 (LKB1) siRNA (sc-270074) and calcium/calmodulin-dependent protein kinase kinase β (CAMKKβ) siRNA (sc-38956) were purchased from Santa Cruz Biotechnologies Inc., CA, USA and were transfected into BM-EPCs using RNAimax (Invitrogen, Carlsbad, CA, 13778) according to manufacturer instructions.

### Functional analysis of BM-EPCs

Effects of pravastatin on Dex-induced EPCs dysfunction was assessed through functional (migration and tube formation) and quantitative methods (*n* = 5 each group).

#### Migration assay

The migratory ability of BM-EPCs was evaluated using a modified Boyden chamber assay. Briefly, 5 × 10^4^ BM-EPCs per plate were placed in the upper chambers of 24-well plate (Corning Transwell, Lowell, MA) with polycarbonate membrane (8 μm pore size) that contained serum-free endothelial growth medium. VEGF (50 ng/ml) was added to medium placed in the lower chambers. After 24 h incubation, the membrane was fixed with 2% paraformaldehyde, and stained by Hoechst 33258 10 μg/ml (Sigma-Aldrich, St. Louis, MO). Five random low-power (×50) microscopic fields per sample were selected to count stained cells that had migrated into the lower chamber (Yin et al., 2007).

#### Tube formation assay

The angiogenic capacity of BM-EPCs was evaluated by the Matrigel tube formation assay. Briefly, 4 × 10^4^ BM-EPCs were plated in 96-well plates that were pre-coated with 50 μl/well growth factor-reduced Matrigel (BD Biosciences, Bedford, MA) and incubated at 37°C for 6 h. Tube formation ability of BM-EPCs was assessed by counting tube number with a computer-assisted microscope (Chen et al., 2013) (Leica Microsystems Inc., Buffalo Grove, IL). Images of tube morphology were taken in 5 random low-power (×50) microscopic fields per sample.

#### Quantification of circulating EPCs

The quantification of circulating EPCs was assessed by flow cytometry. Briefly, 2 mL of peripheral blood was homogenized with 2 ml PBS and then centrifuged at 1500 g for 5 min. Single-cell suspensions were re-suspended in 2 ml PBS and incubated at room temperature for 60 min with anti-Sca-1 and anti-Flk-1 antibody (BD Pharmingen, San Diego, CA, USA). Immunofluorescence was detected using flow cytometry (Becton Dickinson, USA). To quantify circulating EPCs, the number of Sca-1/Flk-1 double-positive cells was counted within the mononuclear cell population.

### Western blot

Total protein was extracted from BM-EPCs using a standard protein extraction kit (KeyGEN Biotechnology; Nanjing, China). Protein was quantified by a BCA Protein Assay Kit (Thermo, Rockford, USA). Samples containing equal amounts of protein were run on 10% SDS-PAGE and subjected to immunoblotting with the following anti-microtubule-associated protein light chain 3II (LC3II, 1:500; Novus Biologicals, NB100-2220); anti-Beclin-1 (3495), anti-LKB1 (3047), anti-p-LKB1 (3482), anti-AMPK (2532), anti-p-AMPK (2535), anti-mTOR (2972), anti-p-mTOR (2971), and CAMKKβ (4436) (1:1000, Cell Signaling Technology, Danvers, Mass); anti-GAPDH (1:5000; Abcam, Cambridge, MA, USA, ab9485). Blots were visualized using Odyssey infrared imaging system (Li-Cor Bioscience, Lincoln, NE), and the bands were quantified by Image-Pro Plus software (Media Cybernetics, Silver Spring, MD). All immunoblotting experiments were repeated at least three times.

### Histopathology

Formalin-fixed decalcified femoral heads of both sides were embedded in paraffin, sectioned at 5 μm, and stained with hematoxylin and eosin (H&E) (Jiang et al., 2015). The sections were evaluated by three independent pathologists in double blind fashion. Femoral head osteonecrosis was defined as the diffuse presence of empty lacunae or pyknotic nuclei of osteocytes in the bone trabeculae, accompanied by surrounding bone marrow cell decrease (Kerachian et al., 2010). Ten high-power fields were randomly chosen, and the ratio of empty lacunae were calculated for each femoral head (*n* = 5 each group).

### Immunohistochemistry

Briefly, paraffin-embedded 5 μm-thick femoral head sections and 4% paraformaldehyde fixed BM-EPCs were incubated with anti-CD31 (1:500, Abcam, Cambridge, MA, USA, ab119339). After being washed three times by PBS, the sections or cells were further incubated with a Vectastain ABC Reagent (Vector, Burlingame, CA, USA). Images were obtained by fluorescence microscope (IX-71, Olympus) with a digital camera (Olympus). Digital images were recorded and analyzed using Image-Pro Plus software (*n* = 5 each group).

### Confocal immunofluorescence microscopy

Cells were fixed with 4% paraformaldehyde for 30 min, permeabilised with 0.2% Triton X-100 (Sigma-Aldrich, T8787) for 10 min, and blocked in PBS containing 5% bovine serum albumin (BSA; Beyotime, ST023) for 30 min. Cells were incubated with anti-LC3 (1:100) at 37°C for 1 h, followed by secondary antibodies conjugated to Alexa Fluor 488. The fluorescence signals were visualized with a TCS SP2 confocal fluorescence microscope (Leica). The Spot Detector BioApplication (Thermo Fisher Scientific) was used to acquire and analyze the images after optimization. Images of 500–1,000 cells for each treatment group were analyzed to obtain the mean LC3 fluorescence punctae number per cell (Tang et al., 2010). (*n* = 5 each group).

### Cell survival assay and lactate dehydrogenase (LDH) release assay

Cell survival was evaluated by a nonradioactive cell counting kit (CCK-8) assay (Dojindo, Kumamoto, CK04-11) according to manufacturer instructions. LDH release analysis was performed with a colorimetric LDH cytotoxicity assay (Promega, G1780). Reaction product was detected by spectrophotometry at 450 nm, using a microtiter plate reader (Tecan).

### Statistical analysis

Data are expressed as mean ± SEM. Differences were evaluated by one-way analysis of variance (ANOVA) followed by Tukey *post-hoc* test (three or more groups). Statistical significance was set at *p* < 0.05.

## Results

### Pravastatin improved histological and MRI outcome of ANFH

**Figure 1:**
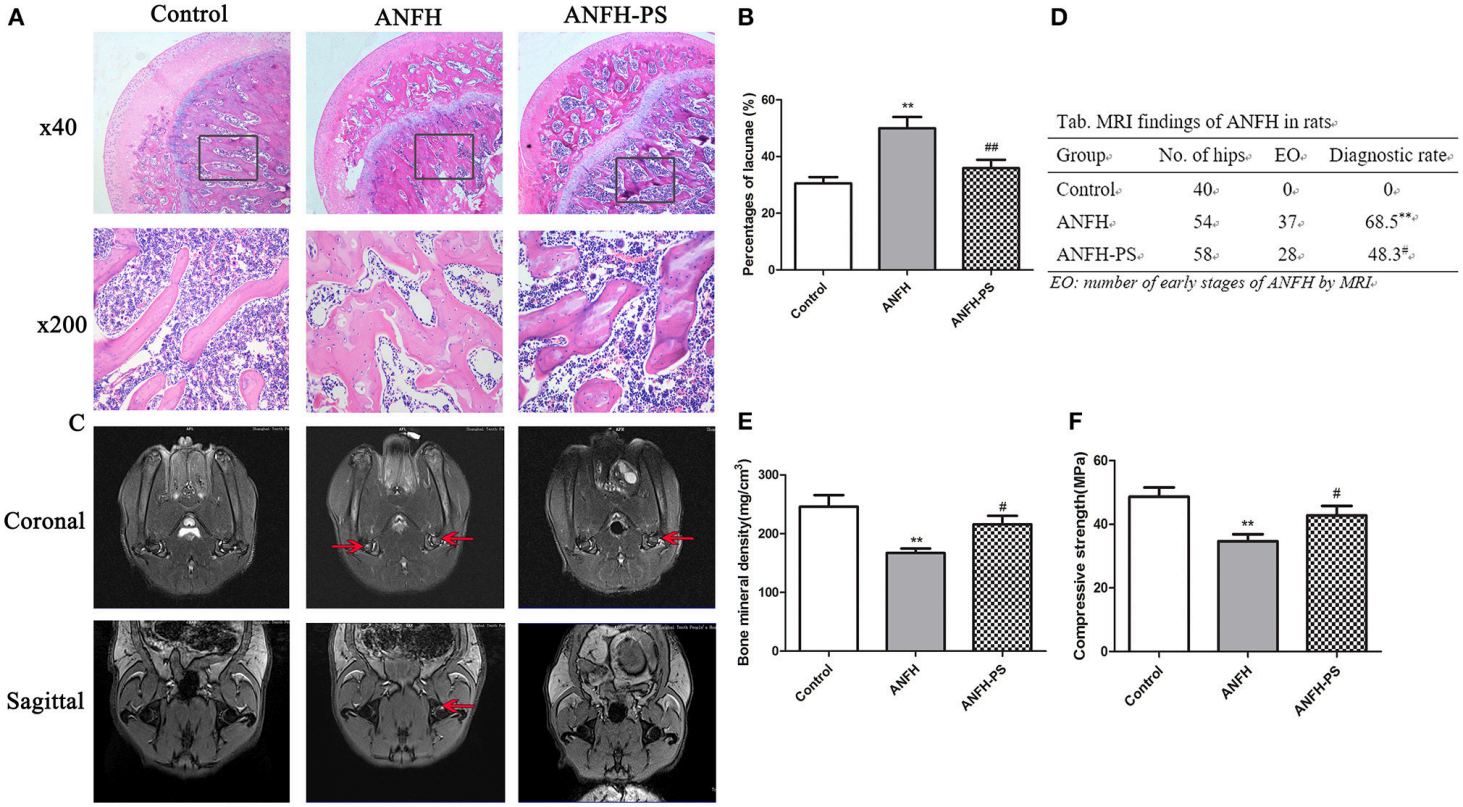
Pravastatin (PS) improved Dex-induced ANFH at histopathological and MRI levels. **(A)** Representative photographs of HE staining on day 30; boxed regions (×40) are shown at higher magnification (×200) below. **(B)** Pravastatin reduced the percentage of empty lacunae in Dex-induced ANFH as compared with the untreated ones. **(C)** Representative photographs of MRI on day 30. The red arrows exhibited STIR high signal which was identified as fat appearance and potential femoral head osteonecrosis. **(D)** The incidence of ANFH by MRI scan in rats. **(E)** Femoral bone mineral density detection. **(F)** Femoral head compressive strength detection. Values are expressed as the mean ± SEM of three independent experiments. *n* = 5 per group. **p < 0.01 vs. Control; ^#^*p* < 0.05, ^##^*p* < 0.01 vs. ANFH.

Osteonecrosis was characterized by empty lacunae, which were visualized histologically following the final application of Dex. Early osteonecrosis was observed in the ANFH group compared to the Control and ANFH-PS (Figures 1A,B). The necrotic area was predominantly located cancellous bone and the subchondral region of the femoral head, adjacent to the femoral neck. The bone marrow displayed a disordered structure, containing necrotic bone marrow cells and aggregated fragments. Bone trabecula appeared thinner, and numerous empty lacunae were observed in ANFH group compared to the Control group. The empty lacunae formations were significantly increased in ANFH rats, compared to that from the Control group (30.61 ± 6.92 vs. 49.97 ± 10.53, *p* < 0.01, Figure 1B), however were significantly decreased following Pravastatin (4 mg/kg) treatment in the ANFH-PS rats (35.92 ± 8.41, *p* < 0.05, Figures 1A,B). Adipogenesis was also largely observed in bone from the ANFH-PS group (Figure 1A).

MRI for ANFH was defined as a geographic area of increased signal on STIR images or decreased signal on T1-weighted images according to the diagnostic criteria (Fujioka et al., 2001). MRI imaging of femoral heads in the Control group displayed a normal level of homogeneity and moderate intensity signal in STIR sequence image (Figure 1C). The MRI image of the ANFH rats displayed abnormality in lipid or myxoid changes and a high signal in STIR. ANFH-PS group demonstrated improvements in imaging compared to the untreated ANFH group, however the flake high signal in STIR was still observable. The incidence of ANFH was calculated in each group according to diagnostic criteria: Control group 0 of 40 femoral heads (0), ANFH group 37 of 54 femoral heads (68.5%), ANFH-PS group 28 of 58 femoral heads (48.3%). A significant difference STIR among the 3 groups (*F* = 45.0, *p* < 0.01, Figure 1D) was observed.

There was significantly lower bone mineral density (167.01 ± 16.07 vs. 245.93 ± 36.49, *p* < 0.01, Figure 1E) and compressive strength (34.68 ± 8.07 vs. 48.67 ± 10.56, *p* < 0.01, Figure 1F) in ANFH rats compared to the Control group. Pravastatin treatment rescued both bone mineral density (215.77 ± 26.37, *p* < 0.05, Figure 1E) and compressive strength (42.76 ± 11.89, *p* < 0.05, Figure 1F) in ANFH-PS rats.

### Pravastatin improved angiogenesis of femoral head and EPCs function and number of ANFH

**Figure 2:**
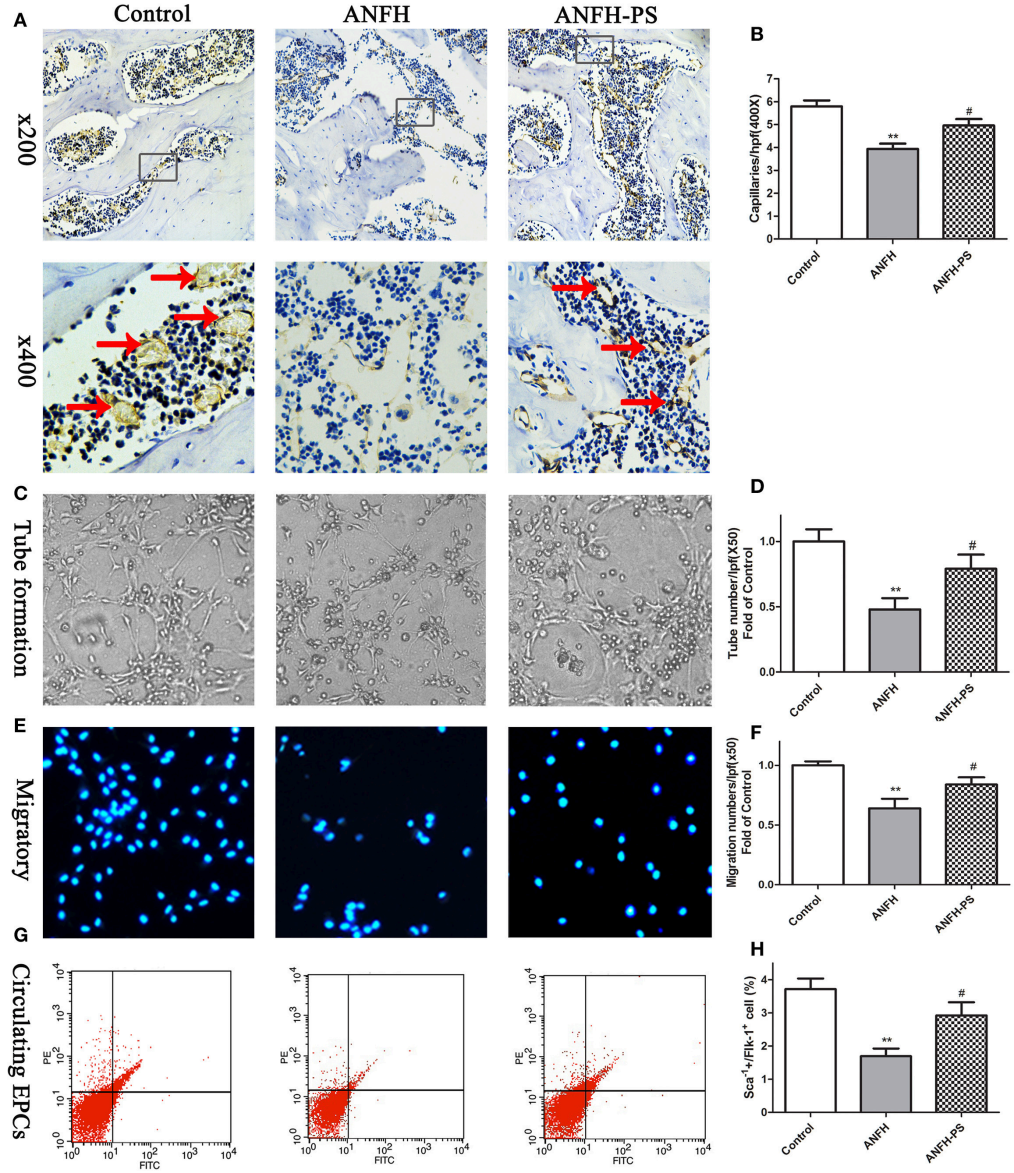
Pravastatin (PS) treatment enhanced femoral head angiogenesis and EPCs functions and numbers in Dex-induced ANFH rats. **(A)** Representative photomicrographs of CD31 staining on day 30; black arrows point to CD31-positive capillaries; boxed regions (×200) are shown at higher magnification (×400) below. **(B)** Quantitative analysis of capillaries per high-power field (hpf) showed that capillaries of femoral head in PS treatment were significantly increased on day 30 when compared with the untreated ANFH ones. **(C,D)** PS enhanced the tube formation capacity on Matrigel of BM-EPCs of ANFH-PS rats. **(E,F)** PS enhanced the migratory capacity of BM-EPCs of ANFH-PS rats. The cells that had migrated into the lower chamber were counted at each group. **(G,H)** PS increased the circulating EPCs number of ANFH-PS rats. Circulating EPCs phenotypes were characterized as these cells co-expressing Sca-1+ and Flk-1+ by fluorescence-activated cell sorting. Values are expressed as the mean ± SEM. *n* = 5 per group. **p < 0.01 vs. Control; ^#^*p* < 0.05 vs. ANFH.

Capillary densities in the femoral head were calculated to evaluate the role of pravastatin on capillary formation in ANFH rats using immunohistochemistry by CD31-specific antibody staining. Following Dex application, the capillary densities in ANFH rats were significantly decreased, compared to the Control group (5.79 ± 0.74 vs. 3.93 ± 0.55, *p* < 0.01, Figures 2A,B). Dex-inhibited capillary density was ameliorated following 30-day pravastatin treatment (4.95 ± 0.64, *p* < 0.05, Figures 2A,B) in ANFH-PS rats.

The capacities of tube formation and migration of BM-EPCs were assessed to determine whether the functions and numbers of EPCs accelerated femoral head angiogenesis in ANFH and ANFH-PS rats. Tubes were defined as a structure exhibiting a length four times its width (Tepper et al., 2002), for analysis of tube formation ability of BM-EPCs by counting tube number with a computer-assisted microscope (Chen et al., 2013). BM-EPCs tube formation (0.47 ± 0.18 vs. 1.00 ± 0.18, *p* < 0.01, Figures 2C,D) and migration (0.63 ± 0.20 vs. 1.00 ± 0.07, *p* < 0.01, Figures 2E,F) capacities from ANFH rats were both significantly lower than Control rats. The percentage of circulating EPCs, characterized as cells co-expressing Sca-1+ and Flk-1+ by fluorescence-activated cell sorting (Feng et al., 2010), was also markedly reduced in ANFH rats, compared to the Control (1.69 ± 0.47 vs. 3.71 ± 0.61, *p* < 0.01, Figures 2G,H). Moreover, pravastatin significantly enhanced tube formation (0.79 ± 0.26, *p* < 0.05, Figures 2C,D) and migration (0.83 ± 0.13, *p* < 0.05, Figures 2E,F) capacities, in addition to restoring reduced numbers of circulating EPCs in ANFH-PS rats (2.92 ± 0.69, *p* < 0.05, Figures 2G,H) compared to ANFH rats. Cultured rat BM-EPCs were also characterized as cells co-expressing Sca-1+ and Flk-1+ by fluorescence-activated cell sorting. Characterization of BM-EPCs was further confirmed as Dil-acLDL and lectin double-positive adherent cells by ?uorescence microscopy (Chen et al., 2013) (Figure S2). Pravastatin did not significantly alter BM-EPCs functions, or circulating EPCs numbers in Control rats (Figure S3).

### Pravastatin regulated BM-EPCs autophagy of ANFH

**Figure 3:**
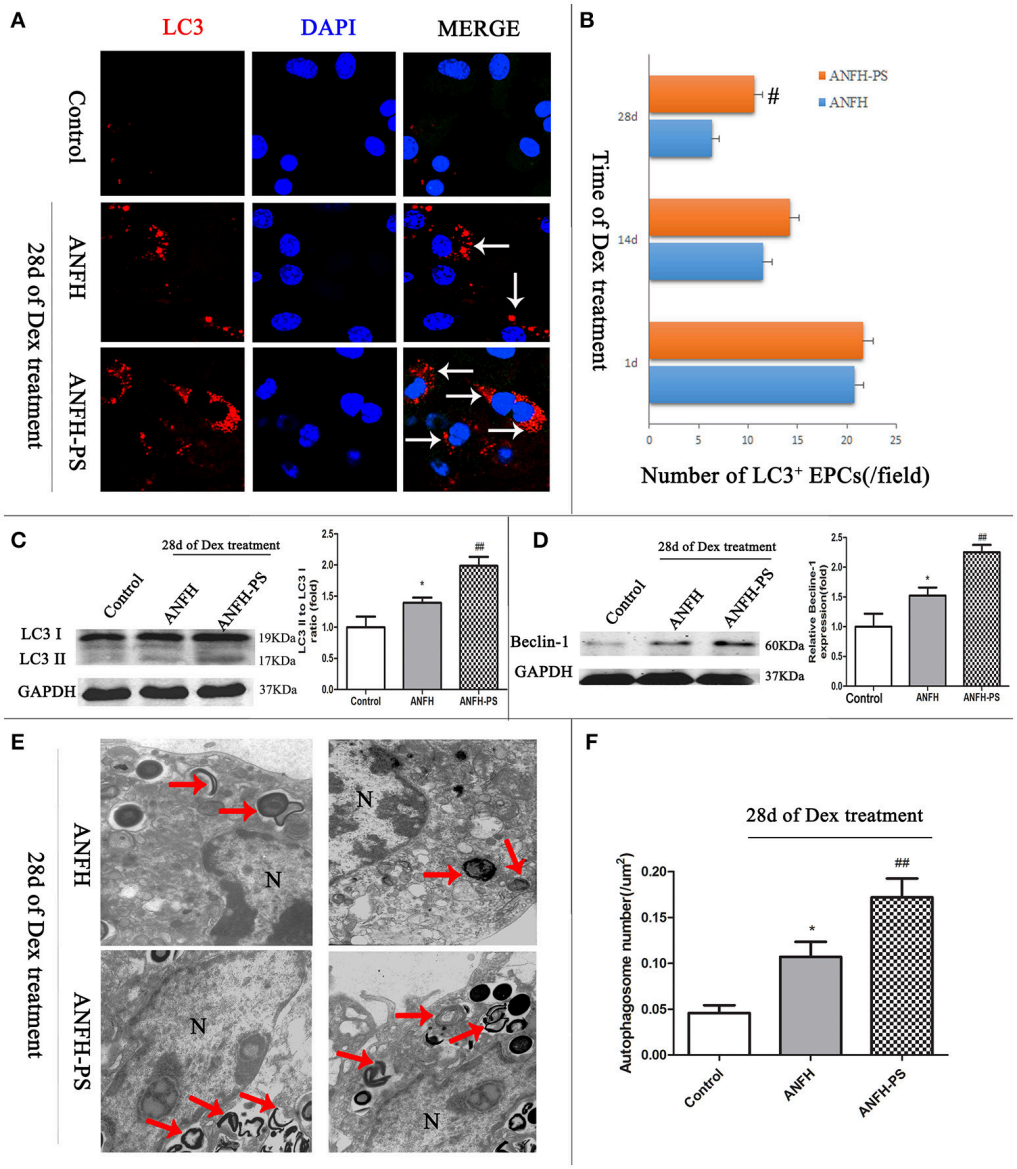
Pravastatin (PS) regulated autophagy of BM-EPCs in Dex-induced ANFH rats. **(A,B)** Immunochemistry and quantitative analysis showed that PS increased the number of LC3^+^ BM-EPCs in ANFH-PS rats. LC3^+^ BM-EPCs were stained in red, the white arrows indicated the LC3-punctate dots. Only the BM-EPCs containing more than 5 LC3-punctate dots were considered to be LC3^+^ BM-EPCs. **(C,D)** Immunoblotting assay showed that PS increased the LC3-II/LC3-I ratio and expression of Beclin-1 in BM-EPCs of ANFH-PS rats. **(E,F)** Representative electron micrographs demonstrated more autophagosomes formation in the ANFH-PS group than ANFH group. The red arrows indicated the double-membraned autophagosomes. N, nucleus. Scale bar, 1 mm. Values are expressed as the mean ± SEM of three independent experiments. *n* = 5 per group. *p < 0.05 vs. Control; ^#^*p* < 0.05, ^##^*p* < 0.01 vs. ANFH.

Microtubule-associated protein light chain 3 (LC3) is the most widely monitored autophagy-related protein and tagged with a fluorescent protein GFP (GFP-LC3) (Klionsky et al., 2016). The percentage of cells with GFP-LC3 puncta was determined by counting the number of positively staining cells, as described in our previous study (Liu et al., 2014). To determine the influence of pravastatin on BM-EPCs autophagy, GFP-LC3 puncta was measured at different time-points (1-, 14-, and 28-days post Dex treatment) by immunohistochemistry (Figures 3A,B). Our data demonstrated that Dex triggered an increase in LC3^+^ puncta BM-EPCs on the first day, however the numbers of LC3^+^ puncta BM-EPCs decreased along with Dex treatment time from 1 day to 28 days in both ANFH and ANFH-PS rats (Figure 3B). Reduced LC3^+^ puncta BM-EPCs were rescued with pravastatin in ANFH-PS rats (10.61 ± 0.90 vs. 6.34 ± 0.82 per field; n = 6, p < 0.01, Figure 3B).

There was constitutive level of LC3 protein in the cytosol as type I (LC3-I) from BM-EPCs. LC3-I can be recruited to autophagosome membrane and converted to LC3-II when autophagy is activated, (Mizushima, 2004). Western blot was utilized to confirm the protein levels of LC3II in BM-EPC. LC3-II was not detected in Control rats, however expression was observed in BM-EPCs of ANFH rats (the ratio of LC3II/LC3I is 1.39 ± 0.25 vs. 1.00 ± 0.47, *p* < 0.01, Figure 3C). Further increase of LC3II/LC3I was observed in ANFH-PS rats (1.98 ± 0.47, p < 0.05, Figure 3C). The expression of Beclin-1 (mammalian homolog of yeast Atg6) was increased in BM-EPCs of ANFH rats compared with Control rats, and further enhanced in ANFH-PS rats (Figure 3D). Autophagosomes in BM-EPCs were monitored by transmission electron microscopy, after the final application of Dex (Figure 3E, red arrow). Transmission electron microscopy analysis revealed apparent nuclear apoptotic phenotype of BM-EPCs in ANFH group, but not in ANFH-PS group (Figure 3E). As expected, the number of autophagic structures in BM-EPCs of ANFH group was higher than that in the Control group (0.09 ± 0.04 vs. 0.04 ± 0.02, *p* < 0.05, Figure 3F), with a significantly increased number of autophagosomes observed in ANFH-PS rats (0.17 ± 0.05, *p* < 0.01, Figure 3F).

### Pravastatin regulated autophagy in cultured BM-EPCs exposed to dexamethasone stress *in vitro*

**Figure 4:**
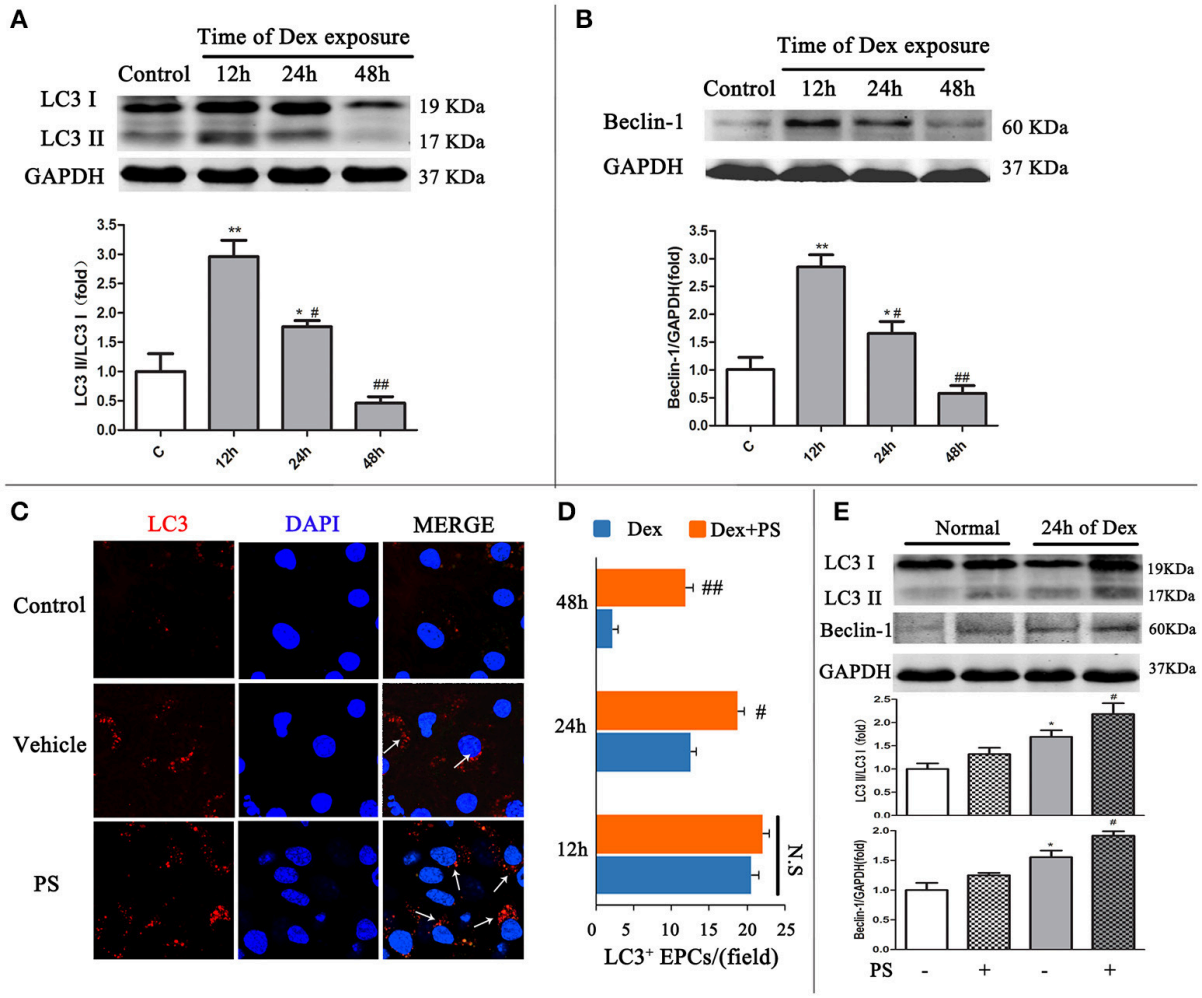
Pravastatin (PS) regulated autophagy BM-EPCs in response to dexamethasone (Dex) stress *in vitro*. **(A,B)** Western blot analysis of LC3II/LC3I and Beclin-1 in BM-EPCs treated with PS (1 uM) under Dex (1 uM) for 12, 24, and 48. The folds of the mean grayscale of LC3II/LC3I, and Beclin-1 to GAPDH among groups are shown. **(C,D)** The representative immunochemistry and quantitative analysis demonstrated that PS began to significantly increased the number of LC3^+^ BM-EPCs at 24 h of Dex exposure. **(E)** Western blot analysis showed that PS enhanced LC3-II/LC3-I ratio and Beclin-1 expression at 24 h of Dex exposure. Values are expressed as the mean ± SEM of three independent experiments. *p < 0.05, **p < 0.01 vs. Control; ^#^*p* < 0.05, ^##^*p* < 0.01 vs. Dex.

Western blot and immunofluorescent staining were used to detect LC3 and Beclin-1, to confirm the effect of Dex-induced BM-EPCs autophagy *in vitro*. Compared with the Control group, the LC3-II/LC3-I ratio in Dex group gradually decreased from 12 h time point (2.95 ± 0.45 vs. 1.00 ± 0.50, *p* < 0.01) to 24 h time point (1.76 ± 0.18, *p* < 0.05), reaching the lowest level at 48 h time point (0.45 ± 0.18, *p* > 0.05, Figure 4A). This data demonstrated autophagy reduction was time-dependent of Dex treatment. Similar pattern was also observed in Beclin-1 in BM-EPCs following Dex exposure (Figure 4B). No significant difference in LC3-positive punctate dots were observed between the Dex-stimulated and pravastatin-treated groups at the 12 h time point (Figures 4C,D). A significantly elevation in LC3-positive punctate dots was observed after pravastatin treatment at the 24 h time point, which increased further at the 48 h time point (Figures 4C,D). Conversely, cell viability dramatically decreased 48 h after Dex stimulation (Figure S4A). The 24 h was therefore selected as the key time point after Dex exposure to observe the effect of pravastatin on autophagy *in vitro*. Pravastatin was further observed to significantly enhanced the LC3-II/LC3-I ratio (2.18 ± 0.43 vs. 1.69 ± 0.23, *p* < 0.05, Figure 4E), along with Beclin-1 (1.91 ± 0.72 vs. 1.55 ± 0.26, *p* < 0.05, Figure 4E) at 24 h after Dex exposure.

### Upregulated autophagy activity contributed to pravastatin mediated BM-EPCs protection

**Figure 5:**
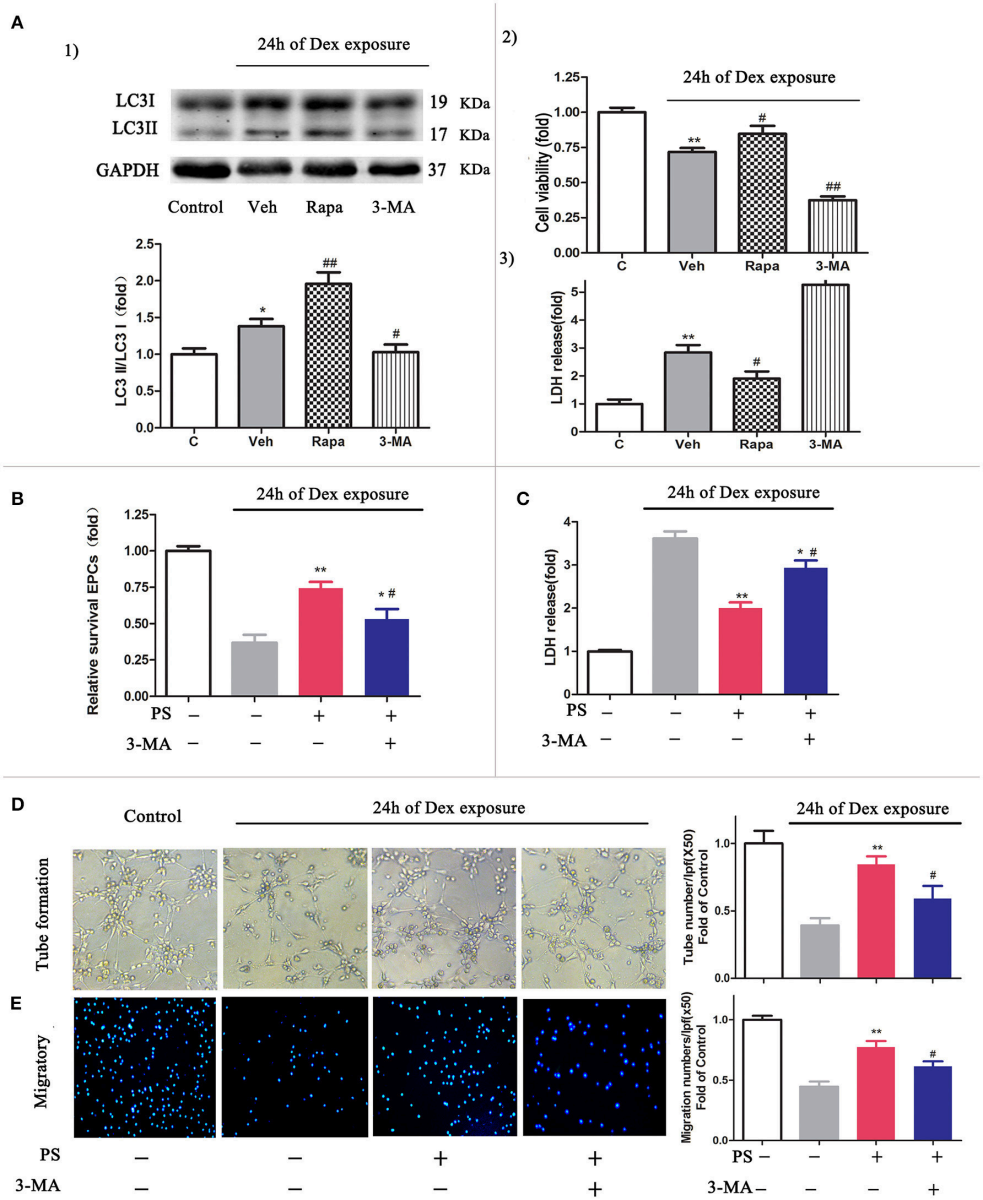
Inhibition autophagy abrogated the protective effects of pravastatin (PS) in BM-EPCs under 24 h of dexamethasone (Dex) exposure. **(A)** Effect of autophagy on the cell viability and LDH release in BM-EPCs exposed to Dex stress. (1) Western blot analysis for the regulatory effect of Rapa (10 nM, autophagy inductor) and 3-MA (5 mM, autophagy inhibitor) on autophagy in BM- EPCs upon Dex (1 μM) stress for 24 h. (2) The cell viability was detected by a CCK-8 assay and 3) LDH release was measured by colorimetric assay. **(B)** 3-MA significantly decreased the viability rate in PS (1 μM)-treated BM-EPCs. **(C)** 3-MA significantly increased the LDH release in PS-treated BM-EPCs. **(D,E)** The protection of PS on BM-EPCs tube formation and migratory capacities were reversed by 3-MA under 24 h of Dex exposure. Values are expressed as the mean ± SEM of three independent experiments. *p < 0.05, **p < 0.01 vs. Control; ^#^*p* < 0.05, ^##^*p* < 0.01 vs. Dex.

To determine whether the activation of autophagy protects cells from Dex-induced cytotoxicity (Liu et al., 2016), we evaluated the cell viability and the level of LDH in the cells following 24 h Dex stimulation, in addition to autophagy promoter rapamycin or autophagy inhibitor 3-MA. The autophagic activity in BM-EPCs was significantly elevated in the presence of rapamycin and Dex, compared with Dex stimulation only (LC3-II/LC3-I ratio 1.96 ± 0.36 vs. 1.38 ± 0.27, *p* < 0.01, Figure 5A.1). Application of 3-MA suppressed the autophagic activity induced by Dex in BM-EPCs (LC3-II/LC3-I ratio 1.02 ± 0.23, *p* < 0.05, Figure 5A.1). Additionally, rapamycin-treated BM-EPCs exhibited higher cell viability and lower levels of LDH, compared with Dex exposure group (Figures 5A.2,A.3). Conversely, the inhibition of autophagy with 3-MA exerted an aggravated injury to BM-EPCs, by significantly decreasing cell viability and increasing levels of LDH expression (Figures 5A.2,**A.3**). To further test the role of autophagy in the protective effect of pravastatin, we exposed BM-EPCs to pravastatin with 3-MA under Dex for 24 h. As shown in Figures 5B,C, 3-MA inhibited the protective effect of pravastatin on BM-EPCs. To determine whether pravastatin-induced regulation of autophagy is associated with the improvement of BM-EPCs functionality, the capacities of migration and tube formation of BM-EPCs were assessed 24 h after Dex exposure. Dex was observed to impair both tube formation (0.39 ± 0.11 vs. 1.00 ± 0.19, *p* < 0.01, Figure 5D) and the capacity of migration (0.44 ± 0.10 vs. 1.00 ± 0.08, *p* < 0.01, Figure 5E) of cultured BM-EPCs. Pravastatin significantly rescued both BM-EPCs tube formation (0.84 ± 0.13 vs. 0.39 ± 0.11, *p* < 0.01, Figure 5D) and migration (0.77 ± 0.11 vs. 0.44 ± 0.10, *p* < 0.01, Figure 5E) dysfunctions caused by Dex. In addition, 3-MA partly blocked the effect of pravastatin on BM-EPCs tube formation (0.59 ± 0.19, *p* < 0.05, Figure 5D) and migration (0.61 ± 0.10, *p* < 0.05, Figure 5E).

### Pravastatin regulated autophagy via the AMPK-mTOR signaling pathway in BM-EPCs upon dexamethasone stress *in vitro*

**Figure 6:**
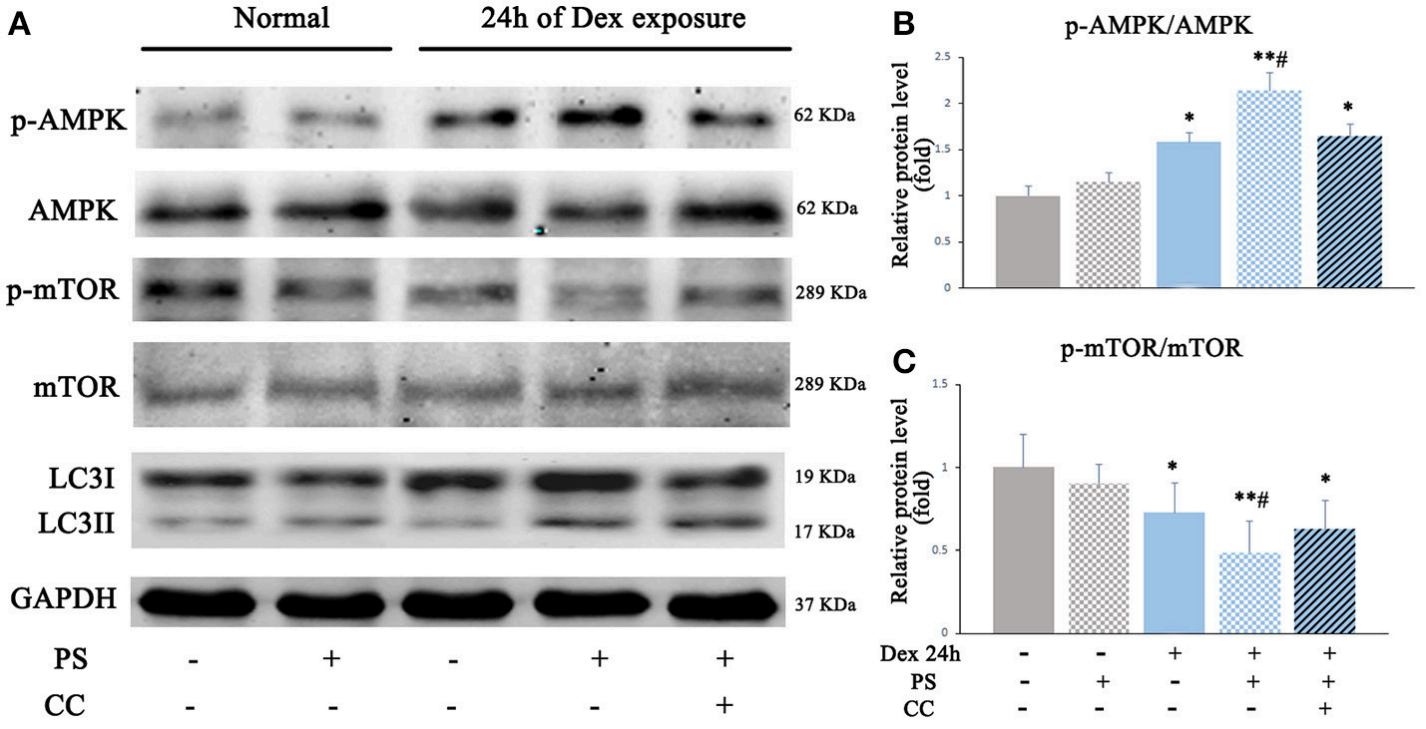
Pravastatin (PS) activated autophagy *via* AMPK-mTOR signaling pathway in BM-EPCs under 24 h of Dex exposure. **(A)** Western blot analysis of p-AMPK/AMPK, p-mTOR/mTOR and LC3II/LC3I in BM-EPCs treated with PS (1 μM) under Dex (1 μM) stress for 24 h **(B,C)**. The quantitative analysis demonstrated that p-AMPK was upregulated and p-mTOR was downregulated in PS-treated BM-EPCs, and inactivation of AMPK by Compound C (10 mM, AMPK inhibitor, CC) resulted in mTOR activation and autophagy inhibition under 24 h of Dex exposure. Values are expressed as the mean ± SEM of three independent experiments. *P < 0.05, **P < 0.01 vs. Control; ^#^*p* < 0.05 vs. Dex.

To investigate whether AMPK-mTOR signaling also mediated the autophagy stimulated by pravastatin, AMPK inhibitor compound C was used and the levels of autophagy were assessed in the pravastatin-treated BM-EPCs under Dex for 24 h. The results revealed that the phosphorylation of AMPK was upregulated (2.13 ± 0.19 vs. 1.58 ± 0.09, *p* < 0.05, Figures 6A,B) while the phosphorylation of mTOR was downregulated (0.48 ± 0.18 vs. 0.72 ± 0.17, *p* < 0.05, Figures 6A,C) in pravastatin-treated BM-EPCs compared with vehicle-only under Dex stress. The phosphorylation of AMPK and mTOR did not change significantly with pravastatin alone. Compound C inhibited the effects of pravastatin on both AMPK phosphorylation (1.64 ± 0.12 vs. 2.13 ± 0.19, *p* < 0.05, Figure 6B) and mTOR phosphorylation (0.62 ± 0.17 vs. 0.48 ± 0.18, *p* < 0.05, Figure 6C). We also observed that pravastatin increased LC3-II/LC3-I ratio (Figure 6A). Conversely, compound C reversed this increase in LC3-II/LC3-I ratio by pravastatin (Figure 6A).

### LKB_1_, but not CaMKKβ, contributed to statin-mediated phosphorylation of AMPK in BM-EPCs upon excess dexamethasone stress (EDS) *in vitro*

**Figure 7:**
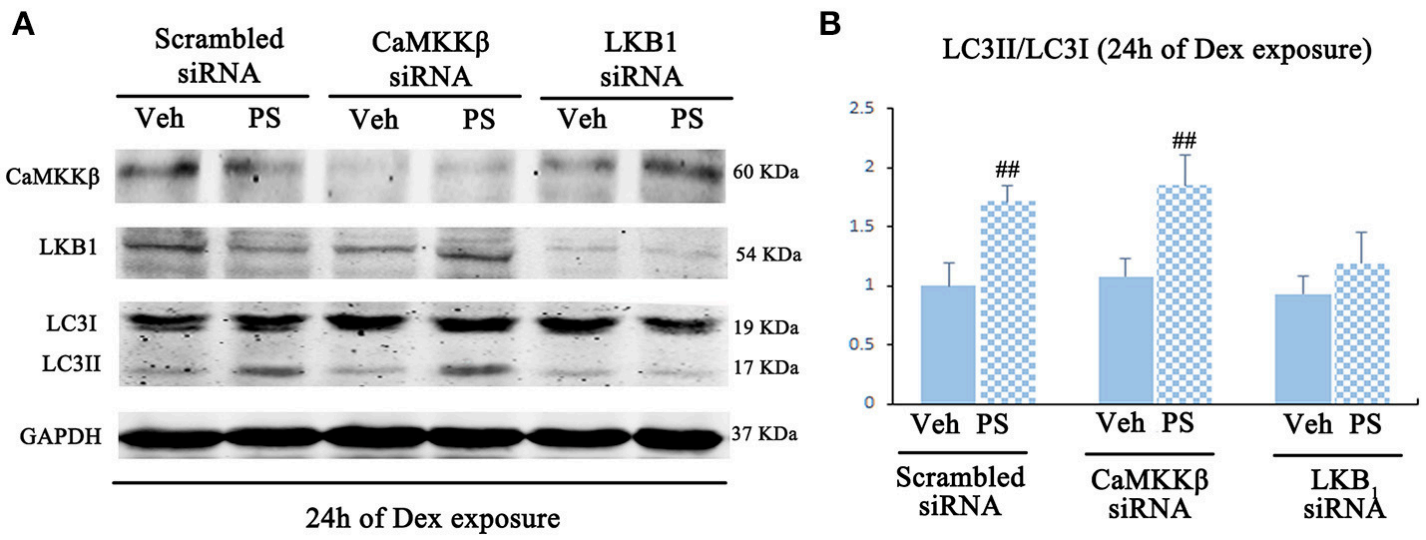
Pravastatin (PS)-induced AMPK activation is mediated by LKB1 in BM-EPCs under 24 h of Dex exposure. **(A)** Western blot analysis for the PS (1 μM)-induced autophagy activation in CaMKKβ or LKB1 knock-downed BM-EPCs under Dex (1 μM) stress for 24 h. **(B)** The quantitative analysis demonstrated that PS-induced autophagy activation was abrogated in LKB1-siRNA BM-EPCs. Values are expressed as the mean ± SEM of three independent experiments. ^##^*p* < 0.01 vs. Dex.

Pravastatin was observed to induce LKB1 phosphorylation in 24 h-Dex stimulated BM-EPCs (Figures S4B,C). LKB1-specific siRNA inhibited the effect of pravastatin on LC3II/LC3I ratio on BM-EPCs *in vitro* (Figure 7). No significant change of pravastatin-induced ratio of LC3II/LC3I following knockdown of CaMKKβ was observed (Figure 7).

## Discussion

Recently, many researchers have become interested in the role of autophagy in osteonecrosis (Xia et al., 2010). Autophagy classically contributes to cellular homeostasis and adaptation to stress, through a complex process that degrades intracellular components by forming autophagosomes, playing a critical role in cellular host defense and cryoprotection (Eisenberg-Lerner et al., 2009). In energy starved or environmental stress conditions, adaptive autophagy degrades, and recycles subcellular organelles, thereby promoting cell survival (Hamacher-Brady et al., 2006). However, dysfunctional autophagy precipitates apoptosis (Azad et al., 2008) and contributes to many diseases including cancer, aging, and degenerative disorders (Ahn et al., 2016; Ward et al., 2016).

In the present study we report that pravastatin ameliorated ANFH by restoring the autophagy process in EPCs. EPCs were used as an indicator for protection of ANFH in animal model. While EPCs are not directly linked to ANFH development, reduced numbers and impaired function of EPCs have previously been correlated with increased risk for ANFH patients (Feng et al., 2010). Our study identified that increased autophagy immediately following Dex stimulation in EPCs was due to cellular self-protection, however this response was gradually lost over longer period of Dex exposure. Concurrently, prolonged Dex exposure was correlated with decreased cell viability, suggesting the activation of autophagy protects cells from Dex-induced cytotoxicity. This is further supported by our cell viability and LDH study *in vitro* whereby 24 h Dex exposure reduced BM-EPCs viability, which was ameliorated when autophagy was induced by rapamycin, a pharmacological autophagy inducer that inhibits mTOR by binding to RPTOR and thus inducing autophagy (Klionsky et al., 2016).

It is therefore reasonable to speculate that GCs attenuate autophagy in BM-EPCs, while increased autophagy partially protects cells from GCs stress. Conversely, when adaptive autophagy is inhibited, cells rapidly progress to necrosis. This hypothesis is in line with other studies (Shen et al., 2015), demonstrating that autophagy protects meniscal cells from GCs-induced apoptosis through the 3-MA inhibitor and by suppressing the phosphorylation of inositol 1,4,5-trisphosphate receptors.

Moreover, our data demonstrated that pravastatin induced BM-EPCs autophagy in a time-dependent manner *in vivo* and *in vitro*. Blockage of autophagy by 3-MA further partially diminished the protective effects of pravastatin on BM-EPCs *in vitro*. Our results are consistent with previous data that demonstrated the protective effects of simvastatin on neuro damage in spinal cord injury *via* Beclin-1 and LC3-II mediated autophagy (Gao et al., 2015). Our data is further in line with findings by Whitehead et al. (2015) that demonstrate significantly improved muscle fatigue in dystrophic mice following the treatment of simvastatin, accompanied with increased level of LC3-II, thereby enhancing autophagic flux (Whitehead et al., 2015). Although we acknowledge the difference between BM-EPCs and neurons, our data demonstrated that pravastatin protected against Dex in BM-EPCs by promoting autophagy activity, and inhibiting autophagy compromised statin-induced EPCs protections.

AMP-activated protein kinase (AMPK) is a key autophagy regulator which is activated under chronic stress or ischemia conditions by sensing cellular energy changes, resulting in decreasing ATP/AMP ratio and indirectly suppressing activation of mTOR. This interrupts mTOR signaling by AMPK enhances autophagy (Yang and Klionsky, 2010). The specific role of pravastatin in the autophagy process remains unclear, therefore warranting further investigation, however a number of implications have been made. Noticeably, atorvastatin activates the AMPK pathway to protect mesenchymal stem cells from hypoxia and serum-free injury (Dong et al., 2011), which may explain the role of statin in autophagy and AMPK-mTOR pathway. Thus, the expression and activation of AMPK-mTOR pathway were investigated to identify the initiating factor for pravastatin-induced autophagy. Interestingly, we identified that pravastatin increased AMPK activation however significantly reduced mTOR activation. Moreover, AMPK inhibitor counteracts mTOR suppression, as well as, reduces pravastatin-induced autophagy in BM-EPCs via LC3 under Dex stress, suggesting that AMPK-mTOR pathway may be important in enhancing pravastatin induced autophagy. Phosphorylation of AMPK involves two upstream kinases LKB1 or CaMKKβ, which were examined to determine the effect of pravastatin on LKB1 expression upon Dex stress. Our results revealed that pravastatin increased the ratio of p-LKB1/LKB1, which was consistent with increased phosphorylation of AMPK. Furthermore, we ascertained that LKB1-deficient BM-EPCs failed to activate LC3II to pravastatin treatment, suggesting that LKB1 is an AMPK upstream kinase for pravastatin-induced autophagosome formation following Dex stimulation. No obvious change of pravastatin-induced autophagy with or without CaMKKβ knockdown was detected, suggesting that CaMKKβ may be redundant for pravastatin-induced autophagosome formation in BM-EPCs in response to Dex stimulation. This data implies that LKB1, not CaMKKβ, mediates pravastatin-induced autophagy activity *via* AMPK phosphorylation signaling pathway. We hypothesize that autophagy-related LKB1-AMPK-mTOR signaling pathway is a potential key process in the development of GCs-induced ANFH.

Our data suggests potential clinical application of pravastatin in ANFH by promoting EPC function and regulating autophagy. Pravastatin has also been implicated in reducing the risk of necrosis of femoral head progression. The underlying mechanism of pravastatin against the development of GCs induced necrosis of the femoral head will be performed through future experiment.

These findings suggest that pravastatin rescues BM-EPCs from Dex-induced autophagy dysfunction *via* AMPK-mTOR signaling pathway in a LKB1-dependent manner. EPCs dysfunction is also suggested to contribute toward the pathogenesis of GCs-induced ANFH. Our results therefore provide insight in the development of novel therapeutics for management of GCs-induced ANFH.

## Ethics statement

All experiments were approved and carried out in accordance to the Shanghai Tenth People's Hospital Ethics Committee (SHDSYY-2015-0018). No human subjective was involved in this experiment.

## Author contributions

YL: designed the experiment, collected and interpreted the data, wrote the manuscript; PZ and BY: collected the data; SB and LL: interpreted the data and reviewed the manuscript.

### Conflict of interest statement

The authors declare that the research was conducted in the absence of any commercial or financial relationships that could be construed as a potential conflict of interest.

## Abbreviations

ANFH: avascular necrosis of the femoral head
ANFH-PS: avascular necrosis of the femoral head and pravastatin
EPCs: endothelial progenitor cells
BM-EPCs: bone Marrow derived-endothelial progenitor cells
GCs: glucocorticoids
Dex: dexamethasone
LC3: microtubule-associated protein light chain 3
AMPK: adenosine monophosphate-activated protein kinase
mTOR: mammalian target of rapamycin
LKB1: liver kinase B1
CaMKKβ: calcium/calmodulin-dependent protein kinase kinase β
MRI: magnetic resonance imaging
STIR: short-tau inversion recovery
LDH: lactate dehydrogenase.
