# Supplementary material for: Pravastatin Protects Against Avascular Necrosis of Femoral Head via Autophagy
Source: Front Physiol. 2018 Apr 9;9:307. doi: 10.3389/fphys.2018.00307 (PMC5900057; doi:10.3389/fphys.2018.00307)
Supplement: Supplementary file 1 [file DataSheet1.PDF]

Title

Pravastatin protects against avascular necrosis of femoral head via autophagy

Yun Liao[1, 2], Ping Zhang[1], Bo Yuan[1], Ling Li[1*], Shisan Bao[1, 3*]

Supplementary information

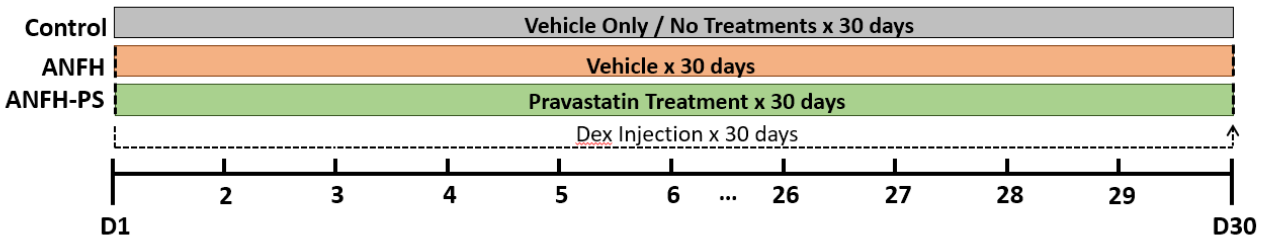

Figure S1. The flow chart of the experiment. Rats in ANFH and ANFH- pravastatin (PS) groups were intravenously injected with Dex (0.5 mg/kg/d×30 d). Meanwhile, the ANFH-PS group were treated over 30 days with PS 4 mg/kg once daily, p.o. On day 30, rats in the three groups were sacrificed, and the femoral heads were harvested or EPCs were isolated from bone marrow.

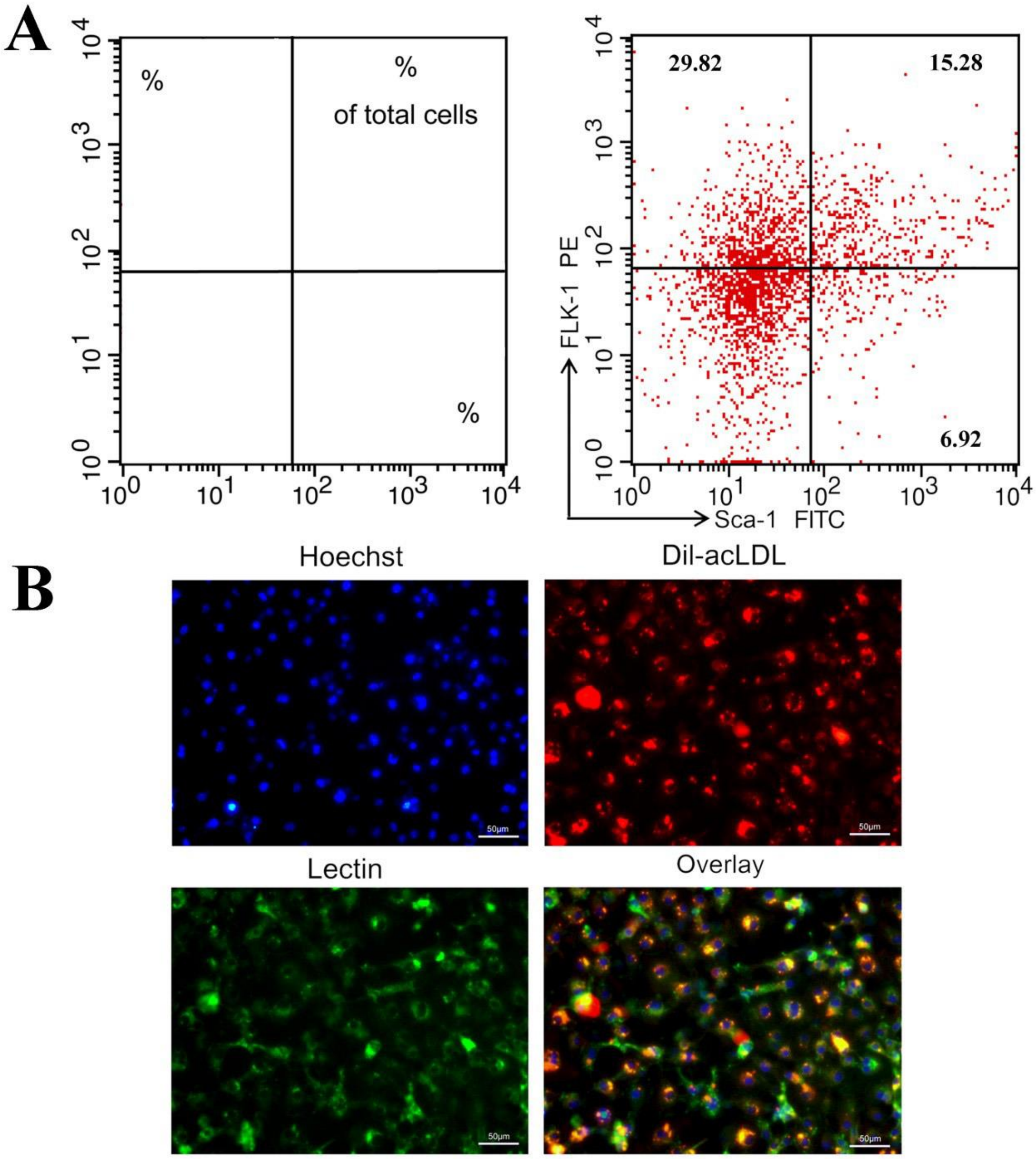

Figure S2. Characterization of rat BM-EPCs. (A) Expression of Sca-1 and Flk-1 in BM-EPCs detected by flow cytometry, double stained cells were defined as BM-EPCs. (B) Double staining of BM-EPCs by Dil-acLDL (red) and Lectin (green). The nuclei were counter-stained with Hoechst (blue). Scale bar: 50 μm. Values are expressed as the mean ± SEM of three independent experiments.

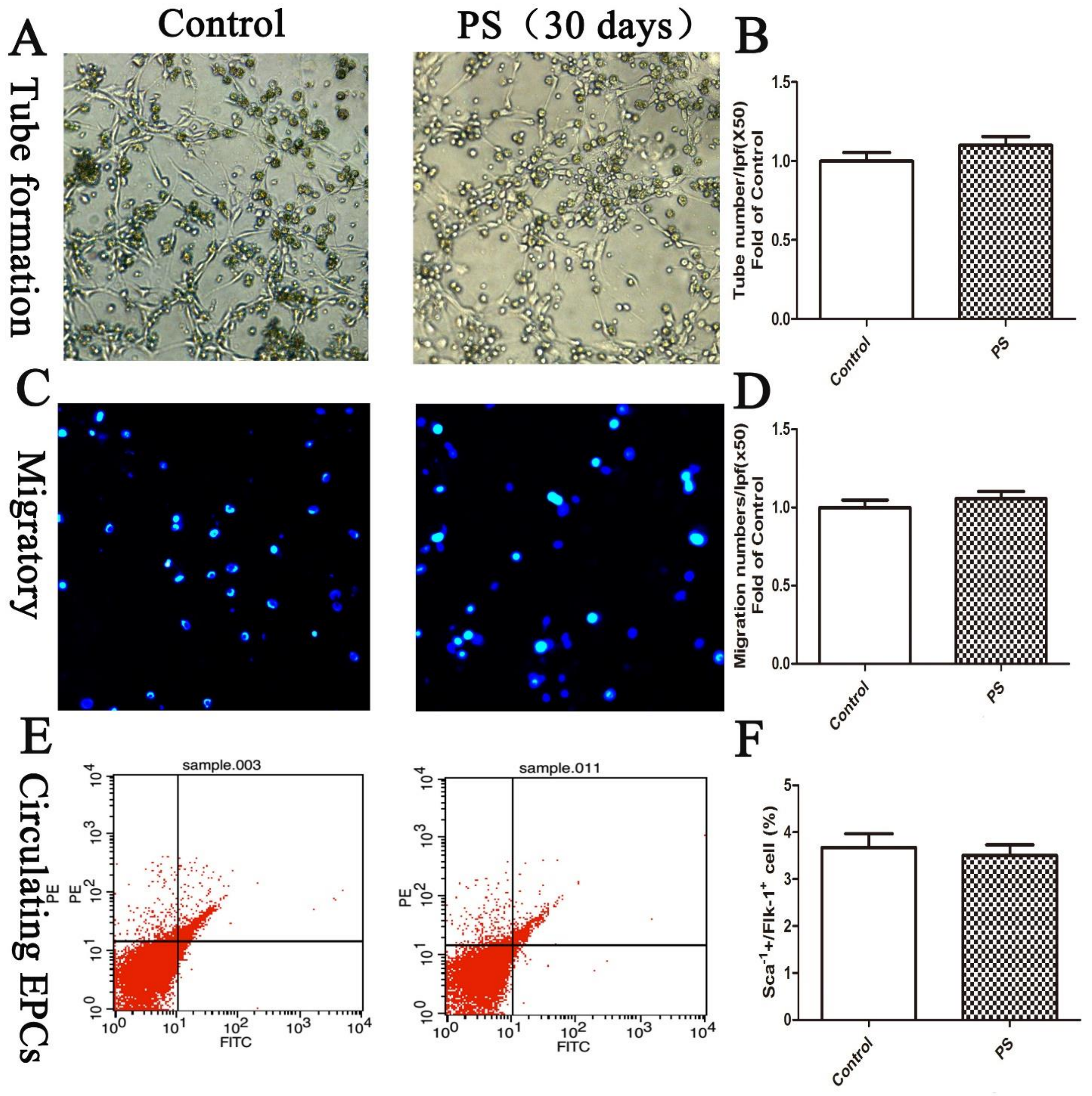

Figure S3. Pravastatin (PS) treatment did not significantly modify BM-EPCs function and circulating EPCs number in rats. (A and B) tube formation capacity, (C and D) migratory capacity, (E and F) circulating EPCs number under normal rats with or without 30 days PS treatment. It was found that PS treatment (4 mg/kg/d×30 d) did not significantly modify BM-EPCs functions and circulating EPCs number in normal rats. Values are expressed as the mean ± SEM of three independent experiments. n= 6-8 per group. *p < 0.05, **p < 0.01 vs Control; #p < 0.05, ##p < 0.01 vs Dex.

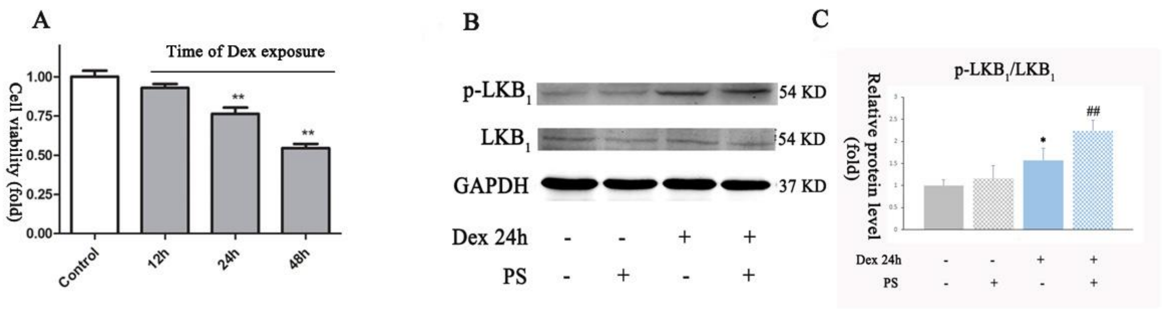

Figure S4. (A) The cell viability was detected by a CCK-8 assay in BM-EPCs treated with pravastatin (PS 1 uM) under Dex (1 uM) for 12 h, 24 h and 48 h. (B) Western blot analysis of p-LKB1/LKB1 in BM-EPCs treated with PS under Dex for 24 h. (C) The quantitative analysis showed that p-LKB1 was upregulated in PS-treated BM-EPCs under Dex for 24 h. Values are expressed as the mean ± SEM of three independent experiments. *$p < 0.05$, **$p < 0.01$ vs Control; #$p < 0.05$, ##$p < 0.01$ vs Dex.
